# Risk factor for steatorrhea in pediatric chronic pancreatitis patients

**DOI:** 10.1186/s12876-018-0902-z

**Published:** 2018-12-05

**Authors:** Lu Hao, Teng Wang, Lin He, Ya-Wei Bi, Di Zhang, Xiang-Peng Zeng, Lei Xin, Jun Pan, Dan Wang, Jun-Tao Ji, Ting-Ting Du, Jin-Huan Lin, Li-Sheng Wang, Wen-Bin Zou, Hui Chen, Ting Xie, Hong-Lei Guo, Bai-Rong Li, Zhuan Liao, Zheng-Lei Xu, Zhao-Shen Li, Liang-Hao Hu

**Affiliations:** 1 Department of Gastroenterology, Hainan Branch of Chinese PLA General Hospital, Hainan, China; 2 Department of Gastroenterology, Gongli Hospital, The Second Military Medical University, Shanghai, China; 3 Department of Gastroenterology, Changhai Hospital, The Second Military Medical University, Shanghai, China; 4 Department of Gastroenterology, The Second Clinical Medical College (Shenzhen People’s Hospital), Jinan University, Guangdong, China; 5 Department of Gastroenterology, Zhongda Hospital, Southeast University, Nanjing, China; 6 Department of Gastroenterology, Air Force General Hospital, Beijing, China

**Keywords:** Chronic pancreatitis, Pediatric, Steatorrhea, Risk factors

## Abstract

**Background:**

Pediatric patients always suffer from chronic pancreatitis (CP), especially those with steatorrhea. This study aimed to identify the incidence of and risk factors for steatorrhea in pediatric CP. To our best knowledge, there is no pediatric study to document the natural history of steatorrhea in CP.

**Methods:**

CP patients admitted to our center from January 2000 to December 2013 were enrolled. Patients were assigned to the pediatric (< 18 years old) and adult group according to their age at onset of CP. Cumulative rates of steatorrhea in both groups were calculated. Risk factors for both groups were identified, respectively.

**Results:**

The median follow-up duration for the whole cohort was 7.6 years. In a total of 2153 patients, 13.5% of them were pediatrics. The mean age at the onset and the diagnosis of CP in pediatrics were 11.622 and 19.727, respectively. Steatorrhea was detected in 46 patients (46/291, 15.8%) in the pediatric group and in 447 patients (447/1862, 24.0%) in the adult group. Age at the onset of CP (hazard ratio [HR], 1.121), diabetes mellitus (DM, HR, 51.140), and severe acute pancreatitis (SAP, HR, 13.946) was identified risk factor for steatorrhea in the pediatric group.

**Conclusions:**

Age at the onset of CP, DM and SAP were identified risk factors for the development of steatorrhea in pediatric CP patients. The high-risk populations were suggested to be followed up closely. They may benefit from a full adequate pancreatic exocrine replacement therapy.

## Background

Chronic pancreatitis (CP) is a rare disease in children. Recent studies have estimated that the incidence of CP in children is approximately 0.5 per 100,000 per year [1–3]. The essence of this disease is the destruction of the organ’s parenchyma by a progressive inflammation process [4]. Pediatric patients with CP always suffer from the severe pain and progressive loss of both exocrine and endocrine function. The irreversible damage of pancreatic exocrine function in CP patients will result in pancreatic enzyme insufficiency (PEI). Severe PEI, or pancreatic exocrine failure, is considered to be clinical steatorrhea, and is a common adverse event of CP. PEI usually manifests as malnutrition, which resulting in vitamin and micronutrient deficiency and weight loss [5, 6], and is at risk of developing premature atherosclerosis, cardiovascular events, osteoporosis, fracture, immune deficiency, and infection [7–9]. PEI is extremely harmful for children. It is well known that malnutrition caused by reduced dietary intake and malabsorption delays the growth and development of these children [10], which also seriously impairs their childhood and mental health [11].

However, in CP patients, a significant proportion of PEI did not show dominant steatorrhea. Functional testing directly for PEI is difficult in clinical practice. Therefore, patients with PEI were rarely confirmed at the early stage [12]. The detection of risk factors for PEI may be clinical important for pediatrics. Pancreatic exocrine replacement therapy (PERT) was recommended in pediatric CP patients according to Australasian Pancreatic Club recommendations [13], but it has a lower level of evidence, and more clinical data was needed. To our best knowledge, there is no pediatric study to document the natural history of steatorrhea in CP. Thus, we aimed to compare the profile of pediatric and adult CP patients. This study was based on a retrospective-prospective cohort of 2153 CP patients with a long duration of follow-up after the onset of CP. We compared the natural history, etiology, complications, and treatment of CP in pediatrics and adults. We also determined the incidence of steatorrhea, and identified the risk factors for this complication in pediatric and adult CP patients, respectively.

## Methods

### Patients and database

The subjects of this study were CP patients hospitalized in Shanghai Changhai Hospital from January 2000 to December 2013. From January 2000 to December 2004, a retrospective collection of patient data was made according to the medical record system, telephone, mail and e-mail follow-up. In order to follow up the patients with CP and facilitate the study of CP. The database system of CP (version 2.1, YINMA Information Technology Company, Shanghai, China) has been established in the Department of Gastroenterology of Changhai Hospital since January 2005 to collect the medical records of patients with CP. Data collected from January 2005 to December 2013 were prospectively collected [12, 14–23]. All patient information is first recorded in a paper-based case report form and then entered into an electronic document. The information collected includes basic information of patients, etiological characteristics (drinking, smoking, anatomic abnormalities, family history), natural course of CP (onset date, onset symptoms, diagnosis date, onset date of pain, pain classification, diagnosis date and treatment history of stones, diabetes mellitus, fatty diarrhea, pseudocysts, common bile duct stenosis); treatment strategy (conservative treatment, endoscopic treatment, surgical treatment).

The database system will remind researchers to notify patients for examination. Except for the examination when patients feel unwell, all patients were checked regularly (at least once a year). Ultrasound, magnetic resonance imaging (MRI), or computed tomography (CT) examination was performed to assess the condition. Patients who did not return to our hospital were followed up by telephone and recorded in the database. The end point of the study was December 2013. In December 2013, we followed up all patients with CP in the database, with the exception of some lost visits and deaths [12]. Follow-up was defined from the onset of CP to the time of the last follow-up, death, or end of follow-up (December 2013), whichever came earliest.

**Fig. 1:**
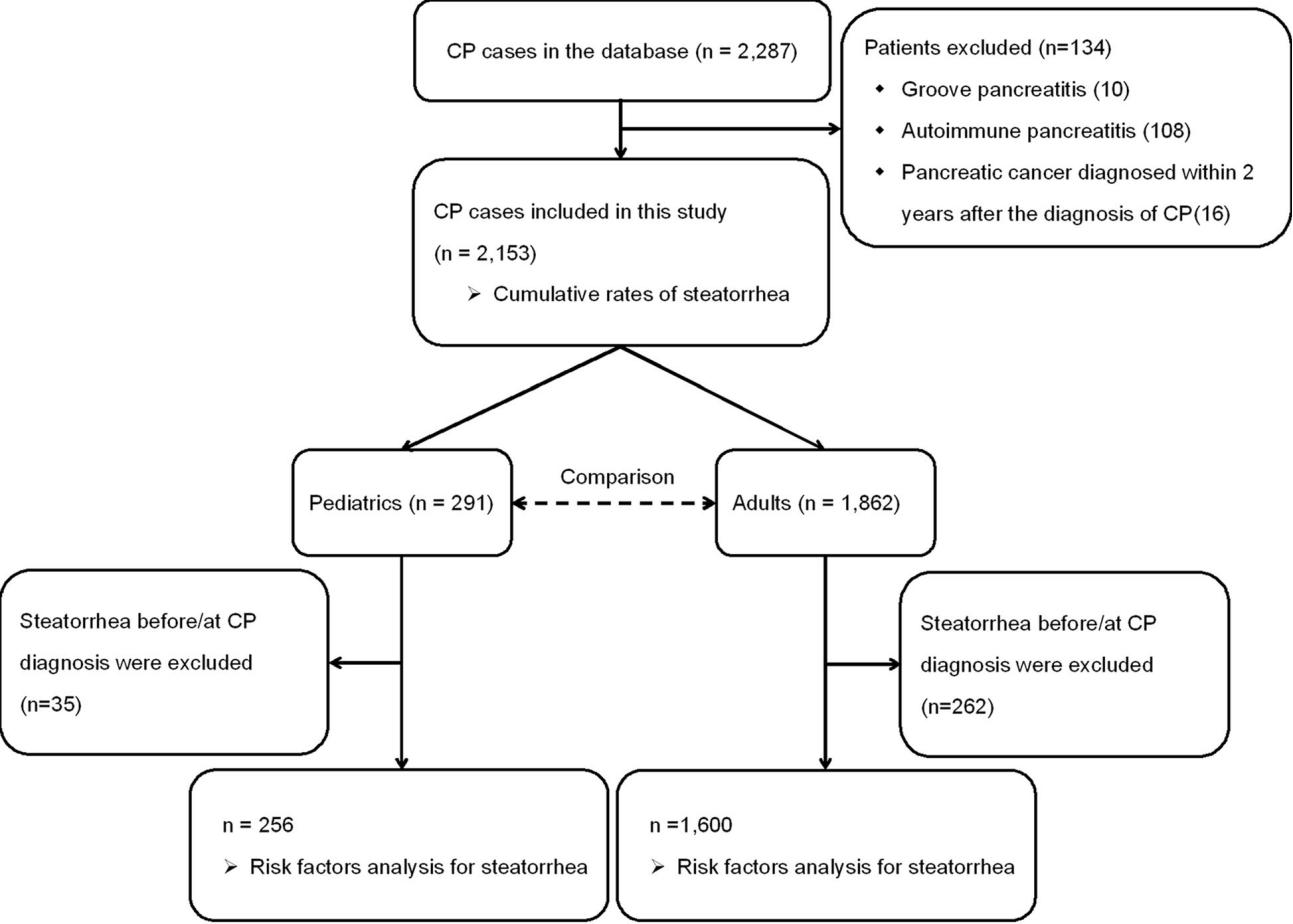
Flow diagram of patient enrolment and the study design

The exclusion criteria for this study were as follows (Figure 1): CP patients diagnosed with pancreatic cancer within 2 years of CP diagnosis [24], grooved pancreatitis (GP) [25], and autoimmune pancreatitis (AIP). Patients were assigned into pediatric group (onset before 18 years of age) and adult group (onset after 18 years of age).

In the study of steatorrhea, patients with steatorrhea diagnosed before CP were excluded in both groups.

The CP database establishing was as mentioned in our previous study [12]. The study was approved by the Ethics Committee of Changhai Hospital. Written informed consent was obtained from all participating patients. All of the diagnostic and therapeutic modalities were carried out in accordance with the approved guidelines.

### Definitions

The diagnosis of CP is based on the 2002 version of Asia Pacific consensus [26]. In the definition of etiologies, men with alcoholic intake of more than 80 g/d or women with alcoholic intake of more than 60 g/d for more than 2 years, excluding other causes, alcoholic CP could be diagnosed [27]. At least 2 first-degree relatives, or at least 3 s-degree relatives with CP and/or recurrent AP, excluding other causes, patients can be diagnosed as hereditary CP [28]. Patients with pancreatic divisum and abnormal pancreaticobiliary drainage are defined as abnormal anatomy of the pancreatic duct (although controversial) [29]. Patients with a clear history of pancreatic trauma and imaging findings suggesting secondary dilatation of the pancreatic duct may be diagnosed as traumatic CP. Hyperlipidemic CP was diagnosed in CP patients with plasma triglyceride > 1000 mg/dL [30]. When all the above causes are excluded, the patient can be diagnosed as idiopathic CP. The definition of severe acute pancreatitis (SAP) was based on the 1992 version of Atlanta classification [31].

Steatorrhea was diagnosed when one of the following two conditions was met: (1) stench, oily chronic diarrhea [32]; (2) positive result in quantification of fecal fat determination (fecal fat quantitation was performed within three days; patients with stool fat excretion over 14 g/day was diagnosed as steatorrhea).

### Treatment strategy

Endoscopic interventional therapy was the first choice for CP patients. Extracorporeal shock wave lithotripsy (ESWL) and endoscopic retrograde cholangiopancreatography (ERCP) were used to remove pancreatic duct stones and drain the main pancreatic duct successfully [15, 33–36]. The indications of surgery in CP patients include: endoscopic interventional therapy can not treat symptoms, combined with CBD stenosis but endoscopic treatment failed, cannot exclude malignant lesions or malignant diagnosed through biopsy, complex conditions and so on [37]. Surgical methods include surgical drainage, pancreaticoduodenectomy and distal pancreatectomy. In painless CP patients, endoscopic intervention or surgical treatment is indicated in patients with CBD stenosis or pancreatic portal hypertension [38]. Indications for endoscopic or surgical treatment did not include simple DM or steatorrhea. The treatment strategies of CP patients were as mentioned in our previous study [12].

### Statistical analysis

In this study, continuous variables are represented in the form of mean ± standard deviation (SD) and compared with an unpaired, 2-tailed *t* test or the Mann-Whitney test. Categorical variables were expressed in the form of counting (percentage) and χ^2^ test or the Fisher exact test were used to compare. CP patients who onset before 18 years of age were assigned into pediatric group and after 18 years of age were assigned into adult groups. The cumulative rates of steatorrhea in both groups after the onset of CP were calculated by Kaplan-Meier method [39]. The statistical analysis were as mentioned in our previous study [12].

Patients who had steatorrhea at/before the diagnosis of CP in pediatric and adult groups were excluded respectively. SPSS (version 21.0) was used to calculate the significance of each variable by multivariate Cox regression analysis in both groups.

## Results

### General characteristics of the subjects

As shown in Figure 1, from January 2000 to December 2013, a total of 2287 CP patients were entered into the Changhai CP Database. After the exclusion of 134 patients, including 10 patients diagnosed with GP, 108 patients diagnosed with AIP, and 16 patients diagnosed with pancreatic cancer within 2 years after the diagnosis of CP, a cohort of 2153 patients with CP was established. The median duration of follow-up was 7.6 years (range 0.0–52.7 years), with 10.4 years (range 0.0–52.7 years) in the pediatrics and 7.0 years (range 0.0–50.0 years) in the adults.

The general characteristics of the patients with CP are presented in Table 1. The mean age at the onset and the diagnosis of CP were 11.622 and 19.727, respectively. The male-to-female ratio in pediatrics was approximately 1:1, while in adults was 3:1. Patients with smoking or drinking history were significantly less in pediatrics (both *P* < 0.001). DM, steatorrhea, pancreatic pseudocyst, and biliary stricture were significantly common in adults (all *P* < 0.05). The etiology and type of pain were also significantly different between the pediatric and the adult groups (both *P* < 0.001).

**Table 1:** General Characteristics of 2153 patients with CP

Items	Overall (*n* = 2153) n (%)	Pediatrics (*n* = 291) n (%)	Adults (*n* = 1862) n (%)	*P* value
Gender (male)	1486 (69.0%)	143 (49.1%)	1343 (72.1%)	< 0.001
Age at the onset of CP, y^a^	38.230 ± 16.606	11.622 ± 4.652	42.388 ± 13.692	< 0.001
Age at the diagnosis of CP, y^a^	43.077 ± 15.548	19.727 ± 8.953	46.727 ± 12.980	< 0.001
Smoking history	723 (33.6%)	16 (5.5%)	707 (38.0%)	< 0.001
Alcohol consumption	–	–	–	< 0.001
0 g/d	1426 (66.2%)	272 (93.5%)	1154 (62.0%)	–
0-20 g/d	70 (3.3%)	8 (2.7%)	62 (3.3%)	–
20-80 g/d	237 (11.0%)	8 (2.7%)	229 (12.3%)	–
> 80 g/d	420 (19.5%)	3 (1.0%)	417 (22.4%)	–
Body mass index^a^	20.894 ± 3.354	19.380 ± 3.362	24.696 ± 88.765	0.338
Etiology	–	–	–	< 0.001
ICP	1633 (75.8%)	248 (85.2%)	1385 (74.4%)	–
ACP	404 (18.8%)	2 (0.7%)	402 (21.6%)	–
Abnormal anatomy of pancreatic duct	64 (3.0%)	24 (8.2%)	40 (2.1%)	–
HCP	30 (1.4%)	12 (4.1%)	18 (1.0%)	–
Post-traumatic CP	10 (0.5%)	3 (1.0%)	7 (0.4%)	–
Hyperlipidemic CP	12 (0.6%)	2 (0.7%)	10 (0.5%)	–
Initial manifestations	–	–	–	< 0.001
Abdominal pain	1796 (83.4%)	280 (96.2%)	1516 (81.4%)	–
Endocrine/Exocrine dysfunction	218 (10.1%)	9 (3.1%)	209 (11.2%)	–
Others	139 (6.5%)	2 (0.7%)	137 (7.4%)	–
Pancreatic stones^b^	1627 (75.6%)	269 (92.4%)	1358 (72.9%)	< 0.001
Age at pancreatic stones diagnosis	41.415 ± 15.323	20.443 ± 8.547	45.569 ± 12.746	< 0.001
Time between onset and pancreatic stone	5.762 ± 7.144	8.829 ± 9.174	5.154 ± 6.504	< 0.001
DM	616 (28.6%)	38 (13.1%)	578 (31.0%)	< 0.001
Age at DM diagnosis^a^	45.848 ± 11.812	28.578 ± 11.965	46.984 ± 10.890	< 0.001
Time between onset and DM^a^	5.136 ± 7.276	16.617 ± 13.447	4.381 ± 5.964	< 0.001
Steatorrhea	493 (22.9%)	46 (15.8%)	447 (24.0%)	0.002
Age at steatorrhea diagnosis^a^	42.563 ± 12.555	25.880 ± 9.358	44.279 ± 11.549	< 0.001
Time between onset and steatorrhea^a^	5.245 ± 8.485	13.929 ± 10.562	4.351 ± 7.719	< 0.001
Pancreatic pseudocyst	350 (16.3%)	30 (10.3%)	320 (17.2%)	0.003
Age at pancreatic pseudocyst diagnosis^a^	45.776 ± 15.077	16.232 ± 7.210	48.589 ± 12.365	< 0.001
Time between onset and pancreatic pseudocyst^a^	4.989 ± 6.954	5.640 ± 5.828	4.927 ± 7.058	0.605
Biliary stricture	340 (15.8%)	19 (6.5%)	321 (17.2%)	< 0.001
Age at biliary stricture diagnosis^a^	51.218 ± 13.169	31.548 ± 13.686	52.382 ± 12.200	< 0.001
Time between onset and biliary stricture^a^	5.592 ± 8.637	21.197 ± 17.565	4.668 ± 6.809	0.001
Pancreatic cancer	21 (1.0%)	1 (0.3%)	20 (1.1%)	0.238
Death	70 (3.3%)	2 (0.7%)	68 (3.7%)	0.008
Morphology of MPD	–	–	–	< 0.001
Pancreatic stone alone	590 (27.4%)	95 (32.6%)	495 (26.6%)	–
MPD stenosis alone	598 (27.8%)	57 (19.6%)	541 (29.1%)	–
MPD stenosis and stone	728 (33.8%)	128 (44.0%)	600 (32.2%)	–
Complex pathologic changes	237 (11.0%)	11 (3.8%)	226 (12.1%)	–
Type of pain	–	–	–	< 0.001
Recurrent acute pancreatitis	681 (31.6%)	102 (35.1%)	579 (31.3%)	–
Recurrent pain	638 (29.6%)	65 (22.3%)	573 (30.8%)	–
Recurrent acute pancreatitis and pain	570 (26.5%)	106 (36.4%)	464 (24.9%)	–
Chronic pain	106 (4.9%)	14 (4.8%)	92 (4.9%)	–
Without pain	158 (7.3%)	4 (1.4%)	154 (8.3%)	–
Severe acute pancreatitis	66 (3.1%)	7 (2.4%)	59 (3.2%)	0.482
Pancreatic duct successful drainage^c^	1930 (89.6%)	255 (87.6%)	1675 (90.0%)	0.216
Overall treatment	–	–	–	< 0.001
Endotherapy alone	1505 (69.9%)	247 (84.9%)	1258 (67.6%)	–
Surgery alone	244 (11.3%)	10 (3.4%)	234 (12.6%)	–
Both endotherapy and surgery	181 (8.4%)	20 (6.9%)	161 (8.6%)	–
Conservative treatment	223 (10.4%)	14 (4.8%)	209 (11.2%)	–
DM in first−/second−/third-degree relatives	135 (6.3%)	38 (13.1%)	97 (5.2%)	< 0.001
Pancreatic diseases in first−/second−/third-degree relatives (excluding hereditary CP)	37 (1.7%)	15 (5.2%)	22 (1.2%)	< 0.001

*CP* chronic pancreatitis, *DM* diabetes mellitus, *ICP* idiopathic chronic pancreatitis, *ACP* alcoholic chronic pancreatitis, *HCP* hereditary chronic pancreatitis, *MPD* main pancreatic duct

^a^Mean ± SD

^b^Pancreatic calcifications were also regarded as stones that are located in branch pancreatic duct or ductulus

^c^Patients with successful MPD drainage are those whose CP was established after ERCP or pancreatic surgery or those who underwent successful MPD drainage during administration when CP diagnosis was established

### Cumulative rates of steatorrhea

**Fig. 2:**
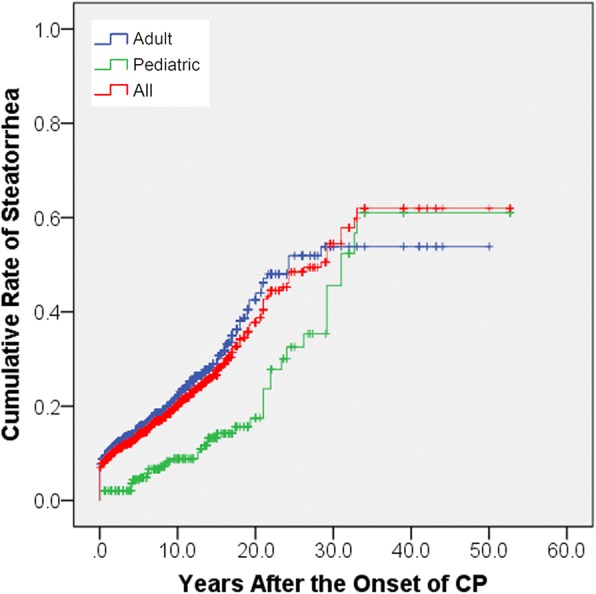
The cumulative rates of steatorrhea after the onset of CP

Steatorrhea was found in 22.9% (493/2153) of patients after the onset of CP. The proportions were 15.8% (46/291) in pediatric patients and 24.0% (447/1862) in adult patients. The cumulative proportions of steatorrhea in pediatric patients were 2.1% (95% confidence interval [CI], 0.5–3.7%), 4.1% (95% CI, 1.6–6.6%) and 7.2% (95% CI, 3.5%-10.9) at 3, 5 and 10 years after the diagnosis of CP, respectively. The cumulative proportions of steatorrhea in adult patients were 12.8% (95% CI, 11.2–14.4%), 14.6% (95% CI, 12.8–16.4%) and 18.3% (95% CI, 16.1–20.5%) at 3, 5 and 10 years after the diagnosis of CP, respectively. Pediatric and adult patients showed significant difference in the rate of steatorrhea (*P* = 0.002, Figure 2).

### Predictors for steatorrhea development in pediatric patients

After the exclusion of 35 patients diagnosed with steatorrhea before the diagnosis of CP in the pediatric patients, a total of 256 patients with CP were finally enrolled in the pediatric group. A univariate analysis for steatorrhea development among the 256 pediatric patients included in the study is shown in Table 2. Three variables showed a *P* value of less than 0.15: age at the onset of CP, DM, and SAP.

**Table 2:** Predictive factors for steatorrhea development in pediatric patients after the diagnosis of CP (256 cases)

Predictors	n (%)	Univariate Analysis		Multivariate Analysis	
		P	HR (95% CI)	P	HR (95% CI)
Gender (male)	124 (48.4%)	0.411	0.353 (0.029–4.233)		
Age at the onset of CP, y^a^	11.573 ± 4.702	0.104	1.121 (0.977–1.286)	0.135	
Age at the diagnosis of CP, y^a^	18.141 ± 6.762	0.235	0.880 (0.712–1.087)		
Smoking history	14 (5.5%)	0.510	4.355 (0.055–346.356)		
Alcohol consumption		0.899			
0 g/d	241 (94.1%)	Control			
0-20 g/d	5 (2.0%)	0.447	0.036 (0.000–2.373E3)		
20-80 g/d	7 (2.7%)	0.716	0.043 (0.000–1.029E6)		
> 80 g/d	3 (1.2%)	0.735	0.042 (0.000–3.846E6)		
Body mass index^a^	19.304 ± 3.338	0.738	0.931 (0.611–1.419)		
Etiology		0.579			
ICP	220 (85.9%)	Control			
ACP	2 (0.8%)	0.710	2.081 (0.043–99.757)		
Abnormal anatomy of pancreatic duct	22 (8.6%)	0.690	2.271 (0.040–127.502)		
HCP	7 (2.7%)	0.912	1.375 (0.005–401.007)		
Post-traumatic CP	3 (1.2%)	1.000	1.008 (0.000–2.361E5)		
Hyperlipidemic CP	2 (0.8%)	0.065	208.297 (0.719–6.036E4)		
Initial manifestations		0.859			
Abdominal pain	249 (97.3%)	0.978	1.392E3 (0.000–9.416E228)		
Endocrine dysfunction	5 (2.0%)	0.972	1.175E4 (0.000–8.352E229)		
Others	2 (0.8%)				
Pancreatic stones^bc^	170 (66.4%)	0.582	1.540 (0.331–7.173)		
Biliary stricture^b^	9 (3.5%)	0.678	0.045 (0.000–1.013E5)		
DM^b^	8 (3.1%)	0.015	51.140 (2.172–1.203E3)	0.806	
Pancreatic pseudocyst^b^	26 (10.2%)	0.762	1.389 (0.165–11.705)		
Morphology of MPD		0.633			
Pancreatic stone alone	82 (32.0%)	0.329	0.082 (0.001–12.473)		
MPD stenosis alone	52 (20.3%)	0.350	0.060 (0.000–21.656)		
MPD stenosis and stone	113 (44.1%)	0.584	0.229 (0.001–44.967)		
Complex pathologic changes	9 (3.5%)	Control			
Type of pain^b^		0.845			
Recurrent acute pancreatitis	93 (36.3%)	0.571	0.218 (0.001–42.016)		
Recurrent pain	48 (18.8%)	0.950	1.167 (0.009–147.028)		
Recurrent acute pancreatitis and pain	92 (35.9%)	0.854	0.637 (0.005–78.045)		
Chronic pain	10 (3.9%)	0.670	0.123 (0.000–1.907E3)		
Without pain	13 (5.1%)	Control			
Severe acute pancreatitis^b^	7 (2.7%)	0.023	13.946 (1.442–134.909)	0.023	13.946 (1.442–134.909)
Pancreatic duct successful drainage^bd^	29 (11.3%)	0.904	0.774 (0.012–50.413)		
Treatment strategy		0.873			
Endotherapy alone	44 (17.2%)	0.876	0.739 (0.017–32.985)		
Surgery alone	11 (4.3%)	0.621	0.231 (0.001–76.658)		
Both endotherapy and surgery	0	0.904	0.774 (0.012–51.413)		
Conservative treatment	201 (78.5%)	Control			
DM in first−/second−/third-degree relatives	29 (11.3%)	0.489	0.042 (0.000–327.986)		
Pancreatic diseases in first−/second−/third-degree relatives (excluding hereditary CP)	12 (4.7%)	0.572	0.278 (0.003–23.531)		

*CP* chronic pancreatitis, *DM* diabetes mellitus, *ICP* idiopathic chronic pancreatitis, *ACP* alcoholic chronic pancreatitis, *HCP* hereditary chronic pancreatitis, *MPD* main pancreatic duct, *HR* hazard ratio, *CI* confidence interval

^a^Mean ± SD

^b^Before or at the diagnosis of CP

^c^Pancreatic calcifications were also regarded as stones that are located in branch pancreatic duct or ductulus

^d^Patients with successful MPD drainage are those whose CP was established after ERCP or pancreatic surgery or those who underwent successful MPD drainage during administration when CP diagnosis was established

For the multivariate analysis, the 3 predictors above were included in the Cox proportional hazards regression model. Finally, 1 predictor for steatorrhea development in pediatric patients was identified. The risk of developing steatorrhea was significantly higher in pediatric patients with a history of SAP before the diagnosis of CP (Hazard ratio [HR], 13.946, 95% CI, 1.442–134.909).

### Predictors for steatorrhea development in adult patients

After the exclusion of 262 patients diagnosed with steatorrhea before the diagnosis of CP in the adult patients, a total of 1600 patients with CP were finally enrolled in the adult group. A univariate analysis for steatorrhea development among the 1600 adult patients included in the study is shown in Table 3. Five variables showed a *P* value of less than 0.05: gender, age at the diagnosis of CP, etiology, initial manifestations, and DM.

**Table 3:** Predictive factors for steatorrhea development in adult patients after the diagnosis of CP (1600 cases)

Predictors	n (%)	Univariate Analysis		Multivariate Analysis	
		P	HR (95%CI)	P	HR (95%CI)
Gender (male)	1161 (72.6%)	< 0.001	2.502 (1.639–3.820)	< 0.001	2.694 (1.756–4.133)
Age at the onset of CP, y^a^	42.777 ± 13.997	0.429	0.996 (0.984–1.007)		
Age at the diagnosis of CP, y^a^	46.798 ± 13.333	< 0.001	0.972 (0.961–0.984)	< 0.001	0.966 (0.953–0.978)
Smoking history	608 (38.0%)	0.188	1.222 (0.907–1.645)		
Alcohol consumption		0.098			
0 g/d	1000 (62.5%)	Control			
0-20 g/d	49 (3.1%)	0.481	0.661 (0.209–2.089)		
20-80 g/d	202 (12.6%)	0.129	1.386 (0.909–2.144)		
> 80 g/d	349 (21.8%)	0.036	1.437 (1.024–2.016)		
Body mass index^a^	25.316 ± 96.332	0.882	0.996 (0.942–1.052)		
Etiology		0.018		0.143	
ICP	1207 (75.4%)	Control		Control	
ACP	338 (21.1%)	0.037	1.414 (1.021–1.956)	0.219	
Abnormal anatomy of pancreatic duct	30 (1.9%)	0.373	0.530 (0.131–2.146)	0.658	
HCP	11 (0.7%)	0.962	0.000 (0.000–3.933E182)	0.345	
Post-traumatic CP	7 (0.4%)	0.003	8.514 (2.088–34.720)	0.041	
Hyperlipidemic CP	7 (0.4%)	0.952	0.000 (0.000–1.191E142)	0.178	
Initial manifestations		< 0.001		< 0.001	
Abdominal pain	1371 (85.7%)	< 0.001	0.401 (0.253–0.636)	< 0.001	0.308 (0.192–0.494)
Endocrine dysfunction	104 (6.5%)	0.130	0.604 (0.315–1.160)	0.059	0.491 (0.235–1.027)
Others	125 (7.8%)	Control		Control	
Pancreatic stones^bc^	1114 (69.6%)	0.830	0.966 (0.701–1.330)		
Biliary stricture^b^	124 (7.8%)	0.097	1.512 (0.928–2.463)		
DM^b^	265 (16.6%)	0.031	1.450 (1.034–2.035)	0.029	1.558 (1.047–2.319)
Pancreatic pseudocyst^b^	123 (7.7%)	0.355	1.284 (0.756–2.180)		
Morphology of MPD		0.063			
Pancreatic stone alone	394 (24.6%)	0.047	1.837 (1.009–3.343)		
MPD stenosis alone	495 (30.9%)	0.016	2.033 (1.144–3.613)		
MPD stenosis and stone	506 (31.6%)	0.194	1.483 (0.818–2.687)		
Complex pathologic changes	205 (12.8%)	Control			
Type of pain^b^		0.086			
Recurrent acute pancreatitis	472 (29.5%)	0.007	0.534 (0.339–0.843)		
Recurrent pain	438 (27.4%)	0.048	0.636 (0.406–0.996)		
Recurrent acute pancreatitis and pain	388 (24.3%)	0.021	0.578 (0.364–0.919)		
Chronic pain	62 (3.9%)	0.206	0.543 (0.211–1.398)		
Without pain	240 (15.0%)	Control			
Severe acute pancreatitis^b^	50 (3.1%)	0.061	0.153 (0.021–1.091)		
Pancreatic duct successful drainage^bd^	223 (13.9%)	0.987	1.004 (0.648–1.555)		
Treatment strategy		0.698			
Endotherapy alone	120 (7.5%)	0.657	0.871 (0.472–1.607)		
Surgery alone	87 (5.4%)	0.282	1.400 (0.758–2.585)		
Both endotherapy and surgery	14 (0.9%)	0.951	0.000 (0.000–3.013E148)		
Conservative treatment	1379 (86.2%)	Control			
DM in first−/second−/third-degree relatives	76 (4.8%)	0.241	0.587 (0.241–1.429)		
Pancreatic diseases in first−/second−/third-degree relatives (excluding hereditary CP)	16 (1.0%)	0.691	0.671 (0.094–4.793)		

*CP* chronic pancreatitis, *DM* diabetes mellitus, *ICP* idiopathic chronic pancreatitis, *ACP* alcoholic chronic pancreatitis, *HCP* hereditary chronic pancreatitis, *MPD* main pancreatic duct, *HR* hazard ratio, *CI* confidence interval

^a^Mean ± SD

^b^Before or at the diagnosis of CP

^c^Pancreatic calcifications were also regarded as stones that are located in branch pancreatic duct or ductulus

^d^Patients with successful MPD drainage are those whose CP was established after ERCP or pancreatic surgery or those who underwent successful MPD drainage during administration when CP diagnosis was established

For the multivariate analysis, the 5 predictors above were included in the Cox proportional hazards regression model. Finally, 4 predictors for steatorrhea development in adult patients were identified. The risk of developing steatorrhea was significantly higher in male patients (HR, 2.694, 95% CI, 1.756–4.133) and patients with a history of DM before the diagnosis of CP (HR, 1.558, 95% CI, 1.047–2.319). Adult patients with an older age at the diagnosis of CP (HR, 0.966, 95% CI, 0.953–0.978) were associated with decreased risk of developing steatorrhea. Initial manifestations were also identified risk factors for steatorrhea development in adult patients.

## Discussion

We focused on CP in pediatrics in the present study. Presence of steatorrhea was set as the sign of severe PEI. To our knowledge, this is the first study to analyze the risk factors of steatorrhea in pediatric patients with CP.

In the present study, 15.8% (46/291) of pediatric patients with CP developed steatorrhea, and 24.0% (447/1862) of adult patients developed steatorrhea. A previous study showed that exocrine and endocrine insufficiency developed more slowly in early-onset CP than in late-onset CP [40]. This could be due to a better preservation of pancreatic function and better repair capacity after injury in pediatric CP patients. However, after a long term of follow-up for more than 30 years, the cumulative rate of steatorrhea in pediatrics was similar or even higher than in adults (Figure 2). Therefore, pediatric CP patients had a reduced risk of steatorrhea compared to adult CP patients in the early period of CP course, but the risk increased with longer-term of follow-up.

In the risk factor analysis, a history of SAP before the diagnosis of CP was identified significantly associated with steatorrhea development in pediatric CP patients. It is not exactly the same as risk factors in adult patients. In adult CP patients, genders, age at the diagnosis of CP, initial manifestations, and DM before the diagnosis of CP were identified risk factors for steatorrhea development. In the previous study, male gender, adults, DM, alcohol abuse and pancreaticoduodenectomy were identified risk factors for steatorrhea development in the general population [12], which are similar with the adult group in the present study.

The risk factor analysis of steatorrhea may help in the early diagnosis of PEI in pediatric patients. Pediatric CP patients with PEI suffer from decreased dietary intake and malabsorption. The malnutrition caused by PEI may retard their growth and development, even result in failure to thrive in these children. This may cause incredible suffering for the children and families who live with them [41]. However, steatorrhea and associated symptoms are not evident until duodenal lipase falls below 5–10% of normal postprandial levels [42]. Thus, PEI may have occurred even the children have no symptoms of steatorrhea. This study provided a relatively accurate risk factor analysis. Age at the onset of CP, DM and SAP were identified the risk factors for steatorrhea in pediatric CP patients. Therefore, these pediatric patient groups were suggested to be closely monitored.

These high-risk populations in pediatric CP patients may benefit from a full adequate PERT. Although PERT was recommended in all pediatric CP patients [13], closely follow-up and dosage adjustment was quite important for these high-risk populations. It can deliver sufficient enzymatic activity into the duodenal lumen simultaneously with the meal, in order to optimize digestion and absorption of nutrients. The PERT will improve the nutritional status for these children and help with their growth and development. This may help in the early treatment of PEI in pediatric patients and reduce the adverse events caused by PEI.

Our study has some limitations. First, clinical steatorrhea was a sign of severe PEI, regardless of dietary habits and steatorrhea associated with abdominal diseases. Second, data was retrospectively collected from 2000 to 2004, which may introduce a recall bias. However, statistical analysis showed that there was no significant difference in clinical characteristics between patients before and after January 2005. In this sense, the recall bias has the least impact on the results. Third, risk factors analysis did not include all potential factors associated with the development of steatorrhea. Fourth, 603 patients with CP were followed up for less than 2 years, which may introduce a misdiagnosis bias between CP and pancreatic cancer.

## Conclusions

In conclusion, steatorrhea is extremely harmful for children. Age at the onset of CP. DM and SAP were identified risk factors for the development of steatorrhea in pediatric CP patients. Therefore, it is suggested that pediatric patients in these high-risk groups be closely followed and examined. They may benefit from adequate PERT.

## Abbreviations

AIP: Autoimmune pancreatitis
CI: Confidence interval
CP: Chronic pancreatitis
CT: Computed tomography
DM: Diabetes mellitus
ERCP: Endoscopic retrograde cholangiopancreatography
ESWL: Extracorporeal shockwave lithotripsy
GP: Groove pancreatitis
HR: Hazard ratio
MRI: Magnetic resonance imaging
PEI: Pancreatic enzyme insufficiency
PERT: Pancreatic exocrine replacement therapy
SAP: Severe acute pancreatitis
SD: Standard deviation
